# Exuberant delayed granulomatous reaction to hyaluronic acid filler material

**DOI:** 10.1093/jscr/rjaf395

**Published:** 2025-06-13

**Authors:** Nasser Almadan, Reem Althubaiti, Chibuzo Uguru, Joseph John K Pothanikat, Jamal Hamed Alshamari

**Affiliations:** Dental Specialist Center, Hafar Al-Batin, Eastern Province 3992, Saudi Arabia; Dental Department, Prince Sultan Military Medical City, Riyadh 12233, Saudi Arabia; Hafr Al-Batin Health Cluster Regional Laboratory Hafr Al-Batin, Eastern Province 39513, Saudi Arabia; Dental Specialist Center, Hafar Al-Batin, Eastern Province 3992, Saudi Arabia; Dental Specialist Center, Hafar Al-Batin, Eastern Province 3992, Saudi Arabia; Dental Specialist Center, Hafar Al-Batin, Eastern Province 3992, Saudi Arabia

**Keywords:** hyaluronic acid, dermal fillers, foreign body granuloma

## Abstract

Dermal fillers are increasing in popularity, especially among young females. Hyaluronic acid is a commonly used injectable material with a relatively safe profile and rare adverse events. In this case, a 34-year-old female patient presented to the oral and maxillofacial clinic in Hafar Al-Batin, Saudi Arabia, with right-sided facial swelling for 1 year. Patient history revealed injection of dermal cosmetic material for 10 years. Magnetic resonance imaging revealed a subcutaneous collection with an irregular wall and adjacent fat stranding. The swelling was removed under general anesthesia, and the tissue was sent for histological diagnosis. The pathological findings included a collection of multinucleated giant cells surrounding cystic spaces filled with frothy, deep blue secretion consistent with foreign body granuloma to hyaluronic acid.

## Introduction

Hyaluronic acid (HA) dermal fillers are the most commonly utilized dermal filler material. It is a non-permanent injectable material that is used to enhance and restore aesthetics [1, 2]. Injection of HA is safe with rare complications with most common reported complication is swelling, bruising, abscess formation, and hyperpigmentation with rare reported cases of granulomatous formation [2–4].

Treatment of HA granulomas includes broad-spectrum antibiotics, systemic steroids, intralesional steroids, or surgical removal [5, 6].

We report a case of delayed granulomatous reaction to HA injection in a 34-year-old female patient who underwent HA injection 10 years ago.

## Case report

**Figure 1:**
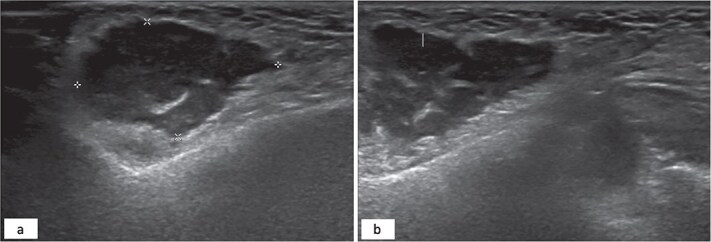
Hypoechoic subcutaneous lesion with an irregular border measuring 3 × 2 cm with internal echoes adjacent to the echogenic fat (a). Internal septation of the hypoechoic lesion (b).

A 34-year-old female patient presented to oral and maxillofacial surgery at the Dental Specialist Center, Hafar Al-Batin, Saudi Arabia, with a complaint of painless right-sided facial swelling for 1 year. The patient reported the injection of a cosmetic filler material 10 years ago. The swelling was a localized nodular and measured approximately 2 × 2 cm, and it was not tender. The patient did not report any pus discharge, and the swelling grew slowly. No gross cranial nerve defects were observed while dental status was good, and no odontogenic etiology was detected. Ultrasound was performed and revealed a subcutaneous collection with internal septa and an irregular wall adjacent to the echogenic fat (Fig. 1).

**Figure 2:**
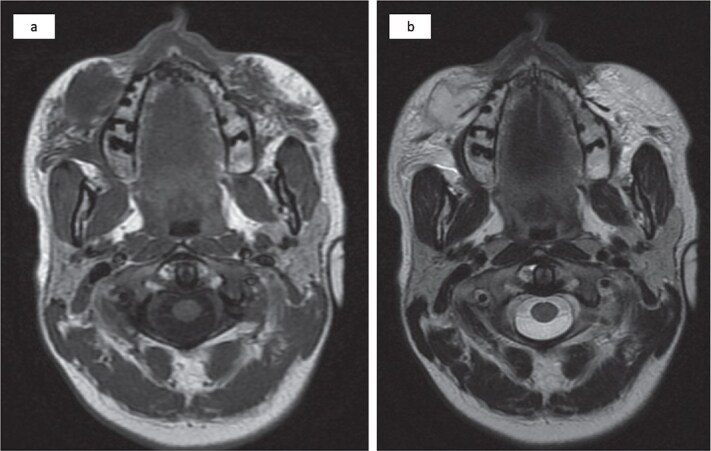
Bilateral injected cosmetic material observed in the subcutaneous tissue of the cheek with the right side showing a subcutaneous hypointense lesion at T1 (a) and a hyperintense lesion at T2 (b) with irregular borders, internal septation, and fat stranding.

Magnetic resonance imaging revealed an injected cosmetic material in the subcutaneous tissue of the cheek on both sides, which was observed in the subcutaneous tissue of the cheek on both sides, with the right side showing a subcutaneous collection with an irregular wall and fat stranding with a low signal at T1 and a high signal at T2 (Fig. 2).

**Figure 3:**
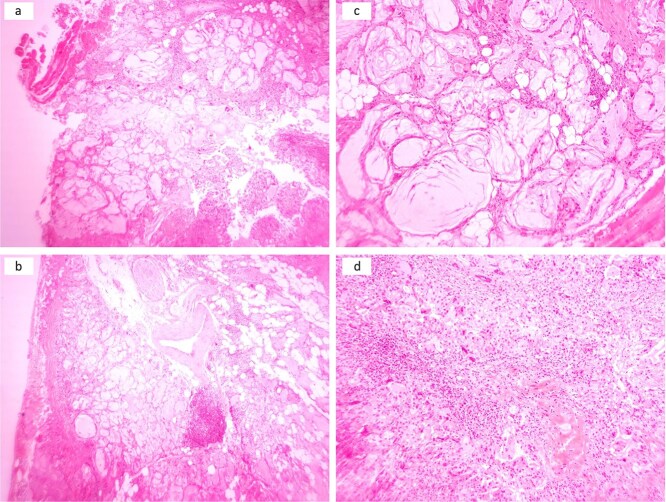
Multiple cystic spaces with amorphous material infiltrating skeletal muscles, adipose tissue, nerves, and large blood vessels with some ruptured spaces leading to exuberant inflammatory reactions (a and b). Cystic spaces with surrounded by palisaded histiocytes and multinucleated giant cells (c). Sheet of histiocytes with scattered multinucleated giant cells and chronic inflammatory cells (d).

The patient underwent enucleation of the lesion under general anesthesia, and the tissue was sent for histopathological examination. It revealed dermal lesion that infiltrated adjacent muscles, nerves, and blood vessels with multiple variably sized cystic spaces with wispy, amorphous, deep blue material with some ruptured cystic spaces, leading to the formation of a non-necrotizing granuloma (Fig. 3).

## Discussion

Delayed granulomatous reaction to HA is a very rare complication and could be related to the non-sterile injection technique, filler material acting as a nidus for infection, a delayed immune response, or an immunological cross-reaction [5]. Some reports have indicated an increase in granulomatous reactions to HA following the COVID-19 pandemic [6].

It is a very rare complication [3, 6, 7]. Clinically, it affects female more commonly, with most cases affecting upper and lower lips followed by cheek and nasolabial fold and it presents as a subcutaneous multiple or single discrete nodule, that is usually painless but could cause tenderness that manifests after injection of HA within 1 year except for one case that developed after 6 years of injection [8]. Histologically, it presents as a variably sized cystic spaces filled with dense amorphous basophilic acellular material surrounded by histiocytes, chronic inflammatory cells, and multinucleated giant cells [3]. Additionally, HA is reactive to alcian blue and colloidal iron special stain while it shows metachromasia to toluidine blue staining and it has diffuse reactivity to CD68 stain [3, 9].

Management of HA granuloma includes the intralesional injection of steroid with the recommended material being triamcinolone. The material is preferably injected from the periphery to center with 1 ml insulin syringe with a 30-gauge needle. Other materials that could be injected includes bleomycin, and 5-fluorouracil. Additionally, antibiotic can be used and the preferred antibiotic are clarithromycin with moxifloxacin, ciprofloxacin, or minocyclin. Recently, hyaluronidase was found to be effective in treating nodule formation related to HA [8]. For recurrent granulomas, systemic steroid therapy can be used with higher dose than the local injection dose [10]. Other treatment approach is the surgical excision; however, this is the last choice as the complete removal is difficult as the granulomas usually invades into surrounding muscle and adipose tissue [7, 10].

## Conclusion

Granulomatous reaction to HA material is a very rare complication to HA dermal filler injection. It is important to consider this differential diagnosis and ask about any history of previous filler injection.
